# Dynamic Programming for Resource Allocation in Multi-Allelic Trait Introgression

**DOI:** 10.3389/fpls.2021.544854

**Published:** 2021-06-18

**Authors:** Ye Han, John N. Cameron, Lizhi Wang, Hieu Pham, William D. Beavis

**Affiliations:** ^1^Department of Industrial and Manufacturing Systems Engineering, Iowa State University, Ames, IA, United States; ^2^Department of Agronomy, Iowa State University, Ames, IA, United States

**Keywords:** dynamic programming, resource allocation, Markov decision processes, plant breeding, multi-allelic trait introgression

## Abstract

Trait introgression is a complex process that plant breeders use to introduce desirable alleles from one variety or species to another. Two of the major types of decisions that must be made during this sophisticated and uncertain workflow are: parental selection and resource allocation. We formulated the trait introgression problem as an engineering process and proposed a Markov Decision Processes (MDP) model to optimize the resource allocation procedure. The efficiency of the MDP model was compared with static resource allocation strategies and their trade-offs among budget, deadline, and probability of success are demonstrated. Simulation results suggest that dynamic resource allocation strategies from the MDP model significantly improve the efficiency of the trait introgression by allocating the right amount of resources according to the genetic outcome of previous generations.

## 1. Introduction

Plant breeding has been defined as the art and science of producing desired characteristics through artificial selection (Poehlman, 2013). Practiced since the beginning of civilizations, plant breeders in the twentieth century made enormous changes to important agronomic traits, e.g., grain yield and pest resistance, of cereal crops (Duvick, 1994; Rincker et al., 2014). It is the plant breeder's job to identify new, genetically-superior crop varieties by “testing” the varieties in multiple environments, then selecting those that perform the best. The intention of this process is to breed specific varieties so that certain phenotypic traits (such as yield, height, weight, pest resistance, etc.) of two individuals can be carried over into its offspring. Historically, identifying the best varieties has been done by trial and error, with breeders testing their experimental varieties in a diverse set of locations and measuring their performance, then selecting the varieties that display the desired characteristics. However, analogously to two humans having children, not all traits can be seen in each child. Due to the inherent randomness in the plant breeding system, this process can take many years to produce the ideal variety and is inefficient, simply due to the number of potential combinations to create and test.

Methods for discovery of genetic variants (alleles) associated with specific phenotypic variants have been developed over the last 25 years and are now routinely applied using “omics” technologies in forward and reverse genetics approaches. These technological advancements have the potential to shorten the time-period required for the integration of desired traits. Because the genetic variants associated with phenotypic variability are distributed unevenly throughout a germplasm collections and breeding populations, it is challenging to combine the most desirable alleles to create improved cultivars. Traditionally, the transfer of a single desirable allele from an inferior cultivar to a superior cultivar is routinely accomplished using marker assisted breeding strategies (Visscher et al., 1996; Frisch et al., 1999; Frisch and Melchinger, 2005; Peng et al., 2014). However, recent developments have demonstrated that the efficiency of these routine processes can be doubled by reframing the objective using principles from operations research (Cameron et al., 2017; Sun et al., 2017; Moeinizade et al., 2019; Xu et al., 2019).

The more complex challenge of aggregating sets consisting of multiple alleles into cultivars with predictable adaptive trait phenotypes will require a specialized breeding strategy to rapidly transfer multiple desirable genetic alleles from a donor individual to an elite recipient individual. In the vernacular of the plant breeder, this is known as multi-allelic trait introgression (MATI) process. The MATI process can be regarded as a decision making system, of which the components are in uncertain states due to the stochastic nature of genetic reconstruction during crop mating. In this process, the plant breeder has the obligation to obtain the available genotypic and phenotypic information, decide parents to breed, allocate resources and fulfill goals. Hospital et al. (2000) demonstrated via simulation that the marker assisted-selection, such as the Marker-based Truncation Selection (MTS) and the QTL Complementation Selection (QCS) could drastically improve the efficiency of parents selection. Recently, De Beukelaer et al. (2015) adapted optimization concepts with heuristics approaches to design a modern and advanced algorithm to solve the gene pyramiding problem. In order to accurately depict this decision making system and optimize the MATI process, a set of mathematical transformations and formulations have been proposed to frame the MATI process as an engineering system (Han et al., 2017). An algorithmic process with mathematical definitions was designed and parental selection was addressed as a key procedure, which can affect the result dramatically. A new metric called the Predicted Cross Value (PCV) with the assistance of genetic markers for parental selection was proposed. The PCV was defined as a quantification metric for any pair of selected breeding parents. Using the metric of PCV, significant improvements with respect to minimizing the cost and amount of time required for successful trait introgression were demonstrated as well as the great potential for further research on MATI process.

As pointed out in Han et al. (2017) and Cameron et al. (2017), in addition to parental selection, resource allocation also plays a crucial role in improving the efficiency of the MATI process. Hospital et al. (2000) discussed similar simulations with fixed population size in each generation but different selection intensity or the number of parents selected. Herein, we expand our discussion on the decision making problem of resource allocation for MATI and improve the breeding strategy by dynamically adjusting the population size for each generation. Resources allocation, as the major topic of this paper, means intelligently determining the population size during the introgression process to efficiently and effectively utilize the resources. Because of the dynamic and uncertain states of the system, we apply the Markov decision processes (MDP) model to frame MATI processes. The MDP model is a technique for solving stochastic sequential decision making problems (Puterman, 2014). The MDP model has been proved to make contributions to various practical decision making projects, such as optimal replacement policy for a motion picture exhibitor (Swami et al., 2001) or the vehicle mix decision in emergency medical service systems (Chong et al., 2015), which share many similarities with MATI processes.

## 2. Materials and Methods

In this section, we cast the MATI process with resource allocation as a Markov decision process model and present a dynamic programming method to solve it. The general idea of this MDP framework is to dynamically simulate and optimize the parent selection, meiosis, gamete production and crossing and other key steps during the trait introgression process. During the simulation, mathematical analysis is applied to adjust parameters to derive the optimal or near optimal decisions. This section covers the flowchart of this engineering process, the necessary mathematical formulations, the detailed discussion on the resource allocation challenge and the MDP model to solve the model.

### 2.1. The MATI Process

**Figure 1:**
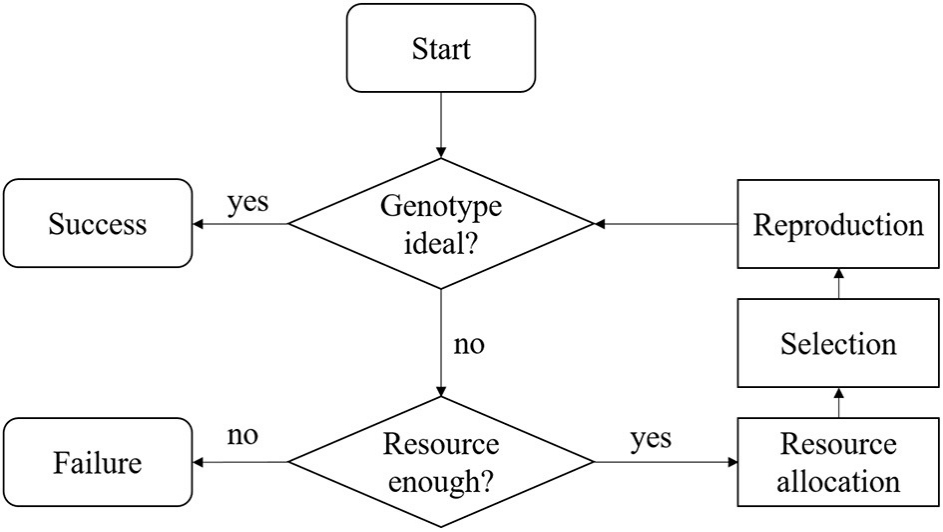
Flowchart of the MATI process.

The work flow for the MATI process is presented in Figure 1. We summarized the MATI process into three steps with two checking points. The three steps are: resources allocation, selection and reproduction, and the two checking points check the available resources and the population genotype.

- The MATI process begins with the **“Start”** step, in which at least one elite recipient individual and one donor individual are available. In most annual crops, both elite and donor individuals are homozygous throughout their genomes. The majority of alleles in the donor are undesirable, but it does have desirable versions of alleles that the elite individual is lacking at several loci. The goal of this process is to achieve an ideal individual inheriting all the desirable alleles from both donor and elite individuals within the provided resources.
- In the **“Genotype ideal?”** check box, the genotypic information of current progeny is screened to check if the ideal individual was produced. If the ideal individual was sampled, the entire process is considered as a **“Success.”**
- Otherwise, the process flows to the **“Resource enough?”** check box. This step involves the resources assessment and the process continues if the remaining resources are adequate. Usually, the resource consists of budget and time. A breeding process is associated with different terms of cost, such as genotyping assays, crossing, growing the crops, and labor. Some costs are fixed, while others are proportional to the number of crosses made or progeny produced. In practice, there may be a total budget constraint for the cost through the entire breeding project. In addition to the cost, the breeding project is often bounded by a deadline, which shall be regarded as a time resource limit.
- In the step **“Resource allocation,”** the decision maker needs to observe the current status of the breeding project and allocate the resources based on policies. For commercial breeding projects, there is revenue associated with the ideal individual when delivered to the market. Hence, for resource allocation, the decision maker needs to consider revenue with the cost.
- When the process reaches the **“Selection”** step, two breeding parents are selected based on a provided selection metric.
- In the **“Reproduction”** step, the selected breeding parents are mated to produce a new generation of progeny and the process flows back to the check box **“Genotype ideal?”** In this MATI process, we assume that the breeding parents would be retained for the next one generation.

### 2.2. Mathematical Formulations for the MATI Process

According to the flowchart, we design a mathematical algorithmic engineering process for simulating the MATI process, in which some steps can be optimized such as **“Resource allocation”** and **“Selection.”** For the **“Selection”** step, random selection, genomic estimated breeding value (GEBV) (Meuwissen et al., 2001), optimal haploid value (OHV) (Daetwyler et al., 2015) and the newly designed predicted cross value (PCV) (Han et al., 2017) are possible metrics for determining the optimal breeding parents for the next generation. For the **“Resource allocation”** step, the remainder of the paper will discuss how to apply dynamic programming model to improve the efficiency. First, we define some major steps in the MATI process.

**Definition 2.1**. (Han et al., 2017) “We define the Reproduce function, *X* = Reproduce(*L*^1^, *L*^2^, *f, K*), as follows. Its input parameters include two binary matrices *L*^1^, *L*^2^ ∈ 𝔹^*N*×2^, a vector *f* ∈ [0, 0.5]^*N*−1^, and a positive integer number *K*. Its output is a three-dimensional matrix *X* ∈ 𝔹^*N*×2×*K*^, representing a random population of *K* progeny.”

The Reproduce function is defined the same way as the one in Han et al. (2017). We use a binary matrix with dimension of *N* × 2 to represent the genotype of a diploid individual with *N* loci where “0” represents undesirable alleles and “1” represents desirable alleles at each of the loci. In the function *L*_1_ and *L*_2_ are the selected breeding parents. The output *X* of the function represents the genotype of all the progeny produced by the breeding parents, whose element *X*_*i*, 1, *k*_ with *i* ∈ {1, 2, …, *N*}, *k* ∈ {1, 2, …, *K*} represents the allele on the *i*th row (locus) of the first set (‘2' on the second dimension of *X* representing the second set) chromosome of the *k*th progeny in the population. The vector *f* ∈ [0, 0.5]^*N*−1^ represents the recombination frequency, which reveals the inheritance characteristics of gene reconstruction. The parameter *K* in the function decides the number of progeny to produce. In the Reproduce function, we assume that the recombination is independent and only related to the recombination frequency.

**Definition 2.2**. We define the Selection function, [*k*_1_, *k*_2_] = Selection(*X*), as follows. Its input parameter includes a three-dimensional binary matrix *X* ∈ 𝔹^*N*×2×*K*^ representing a candidate population. Its output includes two integers, *k*_1_, *k*_2_ ∈ ℤ indicating the indexes of selected parents.

The Reproduce function and the Selection function utilize matrices to represent the information and population genotype. With the information of recombination frequencies, such functions could cast the introgression process into mathematical formulas to be programmed in computer simulation.

**Definition 2.3**. We define the Reward function, Reward(*K, X, t, T*) = Revenue(*X, t, T*) − Cost(*K*), as follows. Its input parameters include a positive integer *K* representing the progeny number, a three-dimensional binary matrix *X* ∈ 𝔹^*N*×2×*K*^ representing a candidate population, a non negative integer *t* representing the current generation number and a non negative integer *T* representing a deadline. Its output is a reward consisting of the revenue from population *X* at generation *t* given deadline *T* and the cost for producing *K* progeny.

**Definition 2.4**. We define the Allocation function,

$$\begin{array}{l} K^{t}=Allocation\left(T,t,f,P^{t},B^{t},Reward\right), \end{array}$$

as follows. Its input parameters include a positive integer *T* representing the deadline, a non negative integer *t* representing the current generation number, a vector *f* ∈ [0, 0.5]^*N*−1^ representing the recombination frequency, a three-dimensional binary matrix *P*^*t*^ ∈ 𝔹^*N*×2×*Kt*−1^ (*t* ≥ 1 and *K*^0^ = 2) representing the candidate breeding population for the current generation (produced by generation *t*−1), a positive number *B*^*t*^ representing the current available budget and the Reward function. Its output *K*^*t*^ is a non negative integer representing the number of progeny to produce for generation *t*. Note that if *K*^*t*^ equals 0 with *t* ≤ *T* and *B*^*t*^ > 0, the project fails.

The Reward function describes the estimated value of certain genotype under assumptions, in relation to current generation and the deadline. This function serves as a measure of quality. Together with the Reward function, the allocation function describes the resources allocation step mathematically. This function determines the population size to produce at a certain generation according to the genetic quality and the time and budget resources left.

With the definitions for three major steps in Flowchart (Figure 1), the definition for simulating the entire MATI process is proposed as follows.

**Definition 2.5**. We define the MATI function, $T_{s}=\text{MATI}\left(P^{0},f,B,\text{Reward},T\right)$, as follows. Its input parameters include a three-dimensional binary matrix *P*^0^ ∈ 𝔹^*N*×2×2^ representing the initial breeding population, a vector *f* ∈ [0, 0.5]^*N*−1^ representing the recombination frequency, a positive integer *B* representing the total budget, a Reward function and a positive integer *T* representing the deadline. Its output *T*_*s*_, is the number of generations the process takes to finish the breeding process, which is determined through the following steps.

**Table:** 

**Step 0 (Initialization)** Set *t* = 0 and go to Step 1.
**Step 1 (Genotype check)**
**If** $\underset{k}{\max}\left\{\overset{N}{\underset{i=1}{\sum }}\left(P_{i,1,k}^{t}+P_{i,2,k}^{t}\right)\right\}=2N$ RETURN: *T*_*s*_ = *t*. **Else** Go to Step 2.
**Step 2 (Resource check and resource allocation)**
*K*^*t*^ = Allocation(*T, t, f, P*^*t*^, *B*^*t*^, Reward). **If** *K*^*t*^ = 0 or *t* > *T* RETURN: *T*_*s*_ = ∞. **Else** Go to Step 3.
**Step 3 (Selection)** Obtain $\left[k_{1}^{t},k_{2}^{t}\right]=\text{Selection}\left(P^{t}\right)$ and go to step 4.
**Step 4 (Reproduction)** Obtain $P^{t+1}=\text{Reproduction}\left(P_{:,:,k_{1}^{t}}^{t},P_{:,:,k_{2}^{t}}^{t},f,K^{t}\right)$, update *t* ← *t*+1 and *B*^*t*+1^ ← *B*^*t*^−Cost(*K*^*t*^), then go to Step 1.

The intuition of the MATI function is as follows:

- Step 0: Initialization;
- Step 1: Check if current population contains the ideal progeny; if it does, return the current generation; otherwise go to the next step;
- Step 2: Check current available time and budget resources and determine the number of progeny to produce; if no resources are left or current time is beyond the deadline, return failure; otherwise go to step 3;
- Step 3: Select the best pair of breeding parents from the current population;
- Step 4: Reproduction step with the determined breeding parents and the number of progeny to produce; Update available resources accordingly; Go back to step 1.

### 2.3. Resource Allocation in the MATI Process

In this section, we propose the problem definition for the resource allocation step in the MATI process, which is related to designing the Allocation function in the MATI function. The resource allocation problem for the MATI process is a dynamic decision making problem. The plant breeder needs to determine how many progeny to produce according to the current generation number, the deadline, the budgets remaining from the total budget, the cost and revenue function and the available progeny at the beginning of each generation. This decision is a key factor affecting the MATI process because it determines the number of offspring produced in each generation as well as the cost and revenue.

Herein, we give some intuitive explanations for the resource allocation problem statement. In each generation, producing more progeny can increase the cost but also the probability of obtaining a more promising genotype. The offspring's genotype and the amount of time together determine the revenue of a project. Generally speaking, the earlier a new genotypically designed product (i.e., offspring) can be delivered to the market, the more market share and revenue a company may attain. Hence, *designing the policy for resource allocation (i.e., how many progeny to produce at each generation) to maximize the expected net present value at the beginning of a breeding project* is regarded as the general problem statement of the resource allocation problem in MATI process.

We frame the resource allocation problem as a dynamic programming problem. Based on the previous discussion, the state describing the status of a breeding project shall consist of genotypic indicators and the budget information. Using metrics like MTS score, QCS score (Hospital et al., 2000) or PCV (Han et al., 2017), we can convert genotypic information into a number and use an interval to cover a group of progeny. Associated with the budget, the state is denoted as a combination of available budget and the metric interval for certain genotypes. By carefully designing the metric intervals, we can make the state space discrete and small enough to enumerate and cover all potential progeny genotypes.

The action that the breeder needs to take is to determine the number of progeny to produce at each state after the evaluation of the available population genotypes, which contains the potential breeding parents for the next generation. This action determines the cost. Meanwhile, different actions affect the probabilities of transitioning among states, which are stored in the transition probabilities matrix. In addition, reaching a specific state at a certain generation will generate revenue. Based on the breeder's estimation, the revenue may not only be decided by the state, but also determined by the current generation number and deadline. There will be a decision policy describing a series of actions to optimize the expected revenue of the breeding project.

In such manners, with a discount factor, the objective of a breeding project can be formulated as determining the optimal policy to maximize the expected net present value in terms of rewards subjected to the deadline and budget. In mathematical formulations, the objective of this resource allocation problem can be stated as:

$$\begin{array}{l} \max_{\pi }{𝔼}_{s}^{\pi }\left\{\sum_{t=0}^{T}\lambda^{t}r_{t}\left(a,s,T\right)\right\}, \end{array}$$

where, *s* represents the state; *a* represents the action; *T* represents the deadline; *r* represents the reward function; λ represents the discount factor and π represents the decision policy.

### 2.4. A Markov Decision Processes Model for Resource Allocation

The dynamic programming structure of the MATI process makes Markov decision processes (MDP) an appropriate approach for solving the stochastic decision making problem. In this section, we formulate an MDP model with finite horizon to identify the optimal resource allocation strategy, which is applied in the Allocation function of the described process.

An MDP model consists of five major components including decision epochs, states, actions, transition probabilities and rewards. The detailed notations for these components are as follows.

**Decision epoches:** We define the **decision epoch** as the beginning of each breeding generation, denoted as {1, 2, 3, …, *T*} and *T* is the deadline of a breeding project. Decisions like parental selection, resource allocation, etc., are made at each decision epoch. We assume the MATI process generally has a specified deadline, which implies that the MDP model has a finite horizon.

**Figure 2:**
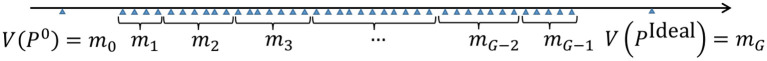
Genotype indicator.

**States:** For any given sample of progeny *P*, we define a function *V*(*P*) to measure the progress in the MATI process, which takes the values within the interval [*V*(*P*^0^), *V*(*P*^Ideal^)], with *P*^0^ and *P*^Ideal^ denoting the original sample of progeny and a sample that includes an ideal individual (with all alleles being desirable). Various definitions of breeding values or parents selection metrics, such as MTS score, QCS score (Hospital et al., 2000) or PCV (Han et al., 2017), could be used for this function. Due to the enormous space of all possible samples of progenies, there is potentially a large number of possible values for *V*. For computational tractability, as illustrated in Figure 2, we group all possible *V* values into a small number of intervals *m*_0_, *m*_1_, *m*_2_, …, *m*_*G*−1_, *m*_*G*_, where *G* is a predetermined integer. In the figure, *m*_0_ is a single value representing the initial population and *m*_*G*_ is another single value representing the final ideal progeny. The intermediate population is represented by each metric interval.

Next we define the **state space** *S* as:

$$S=\left(m_{g},b\right)\cup \left\{\text{failure}\right\}\cup \left\{\text{success}\right\},$$

and

g∈{1,2,…,G-1},b∈{1,2,…,B-1,B},

where (*m*_*g*_, *b*) is a 2-tuple. In the 2-tuple, *m*_*g*_ represents the metric interval indicating the genotype status and *b* represents the remaining budget for the breeding project. In the definition, *B* represents the total budget at the beginning of the process. The design of metric intervals is associated with the preference of the decision maker and shall not be fixed. We will propose one possible approach in the case study section for designing the metric interval. With such state space definition, the initial state is (*m*_0_, *B*)

**Actions:** The **action space** is denoted as $A={⋃}_{s\in S}A_{s}=\left\{0,1,2,\ldots ,a^{\text{max}}\right\}$ representing the number of progeny to produce at each decision epoch. The maximum number of progeny that can be produced is set as *a*^max^ for each generation determined by the reproductive biology of the plant species. In the remainder of this paper, action *a* is used to substitute *K* in the algorithmic process for Allocation function.

**Transition Probabilities:** In the MDP model, we use $W_{i,j}^{a}$ to denote the transition probability from interval *m*_*i*_ in one generation to *m*_*j*_ in the next generation under action *a*. One fact of our MDP model is that once the intervals are determined, *W*^*a*^ only depends on the action *a* and is stationary at different epochs. According to the assumption that the breeding parents are retained to generate a new sample of progeny for the subsequent generation, the process either advances to the next interval or stays in the same one but never moves backwards, i.e., $W_{i,j}^{a}=0$ if *j* < *i*. The matrix *W*^*a*^ could be estimated by simulations recording the information of action, the progeny produced at each generation and the hierarchical kinship information of mating. With the *W*^*a*^ matrix, we are ready to define the transition probabilities matrix, which consists of the probability of transitioning from one state *s* to another state *s*′ under action *a*, i.e., $P_{t}\left(s^{\prime }|s,a\right)$.

**Definition 2.6**. *Given action *a*, the ***transition probabilities matrix*** can be defined as a partitioned matrix *M*^*a*^ as follows*:

$$\begin{array}{l} M^{a}=\begin{array}{l} S_{B}\\ S_{B-1}\\ S_{B-2}\\ \vdots \\ S_{a+1}\\ S_{a}\\ S_{a-1}\\ \vdots \\ S_{1}\\ failure\\ success \end{array}\left(\begin{array}{llllllllll} S_{B}^{⊤} & S_{B-1}^{⊤} & \ldots & S_{B-a}^{⊤} & S_{B-a-1}^{⊤} & S_{B-a-2}^{⊤} & \ldots & S_{1}^{⊤} & failure & success\\ 0 & 0 & \ldots & {\bar{W}}^{a} & 0 & 0 & \ldots & 0 & 0 & {\hat{W}}^{a}\\ 0 & 0 & \ldots & 0 & {\bar{W}}^{a} & 0 & \ldots & 0 & 0 & {\hat{W}}^{a}\\ 0 & 0 & \ldots & 0 & 0 & {\bar{W}}^{a} & \ldots & 0 & 0 & {\hat{W}}^{a}\\ \vdots & \vdots & \ldots & \vdots & \vdots & \vdots & \ddots & \vdots & \vdots & \vdots \\ 0 & 0 & \ldots & 0 & 0 & 0 & \ldots & {\hat{W}}^{a} & 0 & {\hat{W}}^{a}\\ 0 & 0 & \ldots & 0 & 0 & 0 & \ldots & 0 & 1-{\hat{W}}^{a} & {\hat{W}}^{a}\\ 0 & 0 & \ldots & 0 & 0 & 0 & \ldots & 0 & 1 & 0\\ \vdots & \vdots & \ldots & \vdots & \vdots & \vdots & \ldots & \vdots & \vdots & \vdots \\ 0 & 0 & \ldots & 0 & 0 & 0 & \ldots & 0 & 1 & 0\\ 0 & 0 & \ldots & 0 & 0 & 0 & \ldots & 0 & 1 & 0\\ 0 & 0 & \ldots & 0 & 0 & 0 & \ldots & 0 & 0 & 1 \end{array}\right) \end{array}$$

*where* ${\overset{-}{W}}^{a}=W_{1:G-1,1:G-1}^{a}$, ${Ŵ}^{a}=W_{1:G-1,G}^{a}$ *and* $S_{b}={\left[\left(m_{1},b\right),\left(m_{2},b\right),\ldots ,\left(m_{G-2},b\right),\left(m_{G-1},b\right)\right]}^{⊤}$ *is a vector representing* *G*−1 *states. Here*, $P_{t}\left(s^{\prime }|s,a\right)=M_{s,s^{\prime }}^{a},\forall t<T$.

In the definition of the transition probabilities matrix, the matrix ${\overset{-}{W}}^{a}$ represents a sub-matrix containing all the transition probabilities from states group *S*_*b*_ to states group *S*_*b*−*a*_ under action *a*. The vector Ŵ^*a*^ represents a sub-vector containing all the transition probabilities from states group *S*_*b*_ to success under action *a*. Each single value of the transition probability between state *s* and *s*′ under action *a*, which is $P_{t}\left(s^{\prime }|s,a\right)$, is equal to each single element in the matrix $M_{s,s^{\prime }}^{a}$.

**Rewards:** For an MDP model, the reward *r*_*t*_(*s, a*) received at epoch *t* is decided by the state *s* ∈ *S* and action *a* ∈ *A*_*s*_, which can be either positive or negative. In our MDP model for the MATI process, the **reward** is defined as *r*_*t*_(*a, s, T*) = −*C*(*a*) + *R*_*t*_(*s, T*), where *C*(*a*) is the cost function for producing *a* progeny and *R*_*t*_(*s, T*) is the revenue function at epoch *t* associated with state *s* and deadline *T*.

Our finite horizon MDP model can be efficiently solved by the backwards induction method, which is introduced as follows.

**The Backward Induction Algorithm: (Puterman**, **2014****)**

Step 1. Set *t* = *T* and $u_{T}^{*}\left(s\right)=r_{T}\left(s\right)$ for all *s* ∈ *S*.

Step 2. Set *t* ← *t* − 1 for *t* and compute $u_{t}^{*}\left(s_{t}\right)$ for each *s*_*t*_ ∈ *S* by

(1) $$\begin{array}{l} u_{t}^{*}\left(s_{t}\right)=\underset{a\in A_{s_{t}}}{\max}\left\{r_{t}\left(a,s_{t},T\right)+\lambda \underset{s^{\prime }\in S}{\sum }P_{t}\left(s^{\prime }|s_{t},a\right)u_{t+1}^{*}\left(s^{\prime }\right)\right\}. \end{array}$$

and

(2) $$\begin{array}{l} A_{s_{t},t}^{*}=\arg\underset{a\in A_{s_{t}}}{\max}\left\{r_{t}\left(a,s_{t},T\right)+\lambda \underset{s^{\prime }\in S}{\sum }P_{t}\left(s^{\prime }|s_{t},a\right)u_{t+1}^{*}\left(s^{\prime }\right)\right\}. \end{array}$$

Step 3. If *t* = 1, stop. Otherwise return to step 2.

We use π = (*d*_1_, *d*_2_, …, *d*_*T*−1_) to denote a policy, where *d*_*t*_:*S* → *A*_*s*_ is the decision rule prescribing the procedure for action selection in each state at epoch *t*. *r*_*t*_(*a*_*t*_, *s*_*t*_, *T*) denotes the random reward received at epoch *t* < *T* and *r*_*T*_(*s*_*T*_) denotes the terminal reward. $v_{T}^{\pi }\left(s_{1}\right)$ denotes the expected total reward over the decision making horizon if policy π is selected and the system is in state *s*_1_ at the first decision epoch. With the discount factor λ ∈ [0, 1), the expected total discounted reward will be

$$\begin{array}{l} v_{T}^{\pi }\left(s_{1}\right)=E_{s_{1}}^{\pi }\left\{\sum_{t=1}^{T-1}\lambda^{t-1}r_{t}\left(a_{t},s_{t},T\right)+\lambda^{T-1}r_{T}\left(s_{T}\right)\right\}. \end{array}$$

And the total expected reward obtained by using policy π at epochs *t, t* + 1, …, *T* − 1 will be

$$\begin{array}{l} u_{t}^{\pi }\left(s_{t}\right)=E_{s_{t}}^{\pi }\left\{\sum_{n=t}^{T-1}\lambda^{n-1}r_{n}\left(a_{n},s_{n},T\right)+\lambda^{T-1}r_{T}\left(s_{T}\right)\right\}, \end{array}$$

and $u_{T}^{\pi }\left(s_{T}\right)=r_{T}\left(s_{T}\right)$.

Suppose $u_{t}^{*}$, *t* = 1, …, *T* and $A_{s_{t},t}^{*}$, *t* = 1, …, *T* − 1 satisfy equation (1) and (2). Let $d_{t}^{*}\left(s_{t}\right)\in A_{s_{t},t}^{*}$ for all *s*_*t*_ ∈ *S*, *t* = 1, …, *T* − 1 and let $\pi^{*}=\left(d_{1}^{*},\ldots ,d_{T-1}^{*}\right)$. Then, π^*^ is the optimal policy and satisfies

$$\begin{array}{l} v_{T}^{\pi^{*}}\left(s\right)={\text{sup}}_{\pi }v_{T}^{\pi }\left(s\right),s\in S \end{array}$$

and

$$\begin{array}{l} u_{t}^{\pi^{*}}\left(s_{t}\right)=u_{t}^{*}\left(s_{t}\right),s_{t}\in S\text{ for }t=1,\ldots ,T. \end{array}$$

## 3. Results

This section introduces a simulation-based case study for the MDP model to solve the resource allocation problem in MATI process. In this case study, we propose a budget, time and probability of success criteria to assess a breeding strategy. We also discuss how the budget is allocated throughout the process and how to find the most cost-efficient total budget. For purposes of illustrations, we compare static budget allocation strategies and a dynamic budget allocation strategy. All the simulations and case studies are implemented in MATLAB/Octave.

### 3.1. Simulation Setup

We consider a hypothetical project for a case study with the same data structure as the simulation example 1 in Han et al. (2017). As stated in this paper, “We simulated a polygenic trait consisting of 100 markers that are responsible for genetic variability in the trait. The locations of the marker are distributed as uniform random variables among 10 simulated linkage groups. Each linkage group has from 8 to 12 markers. The recipient and donor are homozygous at all QTL. The recipient has desirable markers at 93 loci, while the donor has desirable markers at the remaining 7. For reference, the recipient has undesirable alleles at C1M4, C1M6, C2M9, C3M1, C5M4, C6M3, and C6M8, where C*i*M*j* denotes the *j*th marker in chromosome *i*. Recombination frequencies used in the simulation are given in the Supplementary Materials. The value shown for column C*i* and row M*j* is the recombination frequency between the corresponding marker pairs. The value for adjacent chromosomes is 0.5, in accordance with the principle of independent assortment of chromosomes.” In addition to the genotypic information, Table 1 contains all the parameters for establishing the MDP model. This example represents a realistic plant breeding problem, in which, for instance, 7 disease resistance alleles from a low yield donor need to be introduced to a high yield but disease susceptible recipient. The other 93 markers are used to ensure a high recovery rate of background genes to maintain the favorable agronomic traits of the recipient.

**Table 1:** Parameters.

**Parameter**	**Value**	**Interpretation**
*a*^max^	1,000	maximum progeny number for one generation
*A*	{0, 100, 200, …, 900, 1, 000}	action space
*C*(*a*)	10*a*	cost function
*R*_*t*_(*s, T*)	2, 000, 000−100, 000*t*	nominal market value (revenue) function
*r*_*t*_(*s, T*)	$R_{t}\left(s,T\right)I\left(s=success\right)I\left(t\leq T\right)$	reward function
*T*	8	deadline (in number of generations)
*B*	$11,000, $12,000, …, or $80,000	budget scenarios

Herein, we introduce one possible way to construct the intervals for state space. In order to estimate the intervals, we run 100 preliminary simulations for each possible non-zero action *a* ∈ {100, 200, …, 1000}.

**Table:** 

**Preliminary Simulation:**
**Step 1** Let *P*^0^ denote the initial population and *L*^E^, *L*^D^ denote the elite recipient and donor individuals, respectively, where $P_{:,:,1}^{0}=L^{\text{E}}$ and $P_{:,:,2}^{0}=L^{\text{D}}$.
**Step 2** Set *G* = 0, which represents the current largest terminal generation number.
**Step 3** Set $m_{0}=\text{PCV}\left(L^{\text{E}},L^{\text{D}},f\right)$, in which *f* represents the recombination frequency.
**Step 4**
**For** *a* = 100:100:1000
**For** *n* = 1:100
*g* = 0
**While** $\underset{k}{\max}\left\{\overset{N}{\underset{i=1}{\sum }}\left(P_{i,1,k}^{g}+P_{i,2,k}^{g}\right)\right\}<2N$
$\left[k_{1}^{g},k_{2}^{g}\right]={\mathrm{argmax}}_{k_{1},k_{2}}\left\{\text{PCV}\left(P_{:,:,k_{1}}^{g},P_{:,:,k_{2}}^{g},f\right)\right\}$
$p_{g}^{n,a}=\text{PCV}\left(P_{:,:,k_{1}^{g}}^{g},P_{:,:,k_{2}^{g}}^{g},f\right)$
$P^{g+1}=\text{reproduce}\left(P_{:,:,k_{1}^{g}}^{g},P_{:,:,k_{2}^{g}}^{g},f,a\right)$
*g* = *g*+1
*G* = max(*G, g*)

The intuition of this preliminary simulation is as follows:

- Step 1: Initiate the starting population with the donor and elite recipient; Herein, $P_{:,:,1}^{0}=L^{\text{E}}$ denotes that in the 3-dimensional matrix *P*^0^, all elements in the first and second dimensions are equal to the elite recipient *L*^E^, respectively; The “:” represents all elements in a dimension;
- Step 2: Initiate the current largest generation to achieve the ideal target, which is 0;
- Step 3: Initiate the starting metric point as the PCV value of the donor and elite recipient, with the given recombination frequency;
- Step 4: The major simulation step, simulates the effects of different actions (i.e., different population sizes per generation), on the largest number of generations needed to achieve the ideal target.

In this preliminary simulation, we update the *G* and record the $p_{g}^{n,a}$ for each simulation run. Then, we construct the state space based on the *G* and each $p_{g}^{n,a}$. Since F1 will be the only possible outcome after generation 1, we set $m_{1}=p_{1}^{n,a},\forall n,a$. Similarly, for the last generation *G*, *m*_*G*_ will be the PCV value of the ideal individual, which means $m_{G}=p_{G}^{n,a}=\text{PCV}\left(L^{\text{Ideal}},L^{\text{Ideal}},f\right)$. After the preliminary simulations, we define the interval *m*_*g*_ as $m_{g}=\left[\min_{n,a}\left(p_{g}^{n,a}\right),\min_{n,a}\left(p_{g+1}^{n,a}\right)\right]$ where 2 ≤ *g* ≤ *G-1, n* ∈ {1, …, 100}, *a* ∈ {100, 200, …, 1000}. The state space construction will be trivial based on the definition.

Next, we need to estimate the matrix *W*^*a*^ for the transition probabilities between each state. First, for any given *p*, we can trace back the unique interval that *p* belongs to, based on the preliminary simulation. We use an indicator function *m*_*k*_ = Interval(*p*) to represent this procedure. Meanwhile, we use another matrix *N*^*a*^ ∈ 𝕀^*G*×*G*^ to record the number of simulation runs, which lead to the transition between two intervals under action *a*.

**Table:** 

**For** *a* = 100:100:1000
**For** *n* = 1:100
*g* = 1
**While** $p_{g}^{n,a}<m_{G}$
$m_{k_{1}}=\text{Interval}\left(p_{g}^{n,a}\right)$
$m_{k_{2}}=\text{Interval}\left(p_{g+1}^{n,a}\right)$
$N_{k_{1},k_{2}}^{a}=N_{k_{1},k_{2}}^{a}+1$
*g* = *g*+1
$W_{i,j}^{a}=\frac{N_{i,j}^{a}}{\sum_{j}N_{i,j}^{a}}$

The procedures above introduce how to derive each element in the matrix *N*^*a*^ and how to calculate the transition matrix *W*^*a*^ based on *N*^*a*^.

### 3.2. Simulation Results

We demonstrate the effectiveness of the dynamic programming method for resource allocation by summarizing the results from the simulation experiments.

#### 3.2.1. Tradeoff Among Cost, Time, and Probability of Success

**Figure 3:**
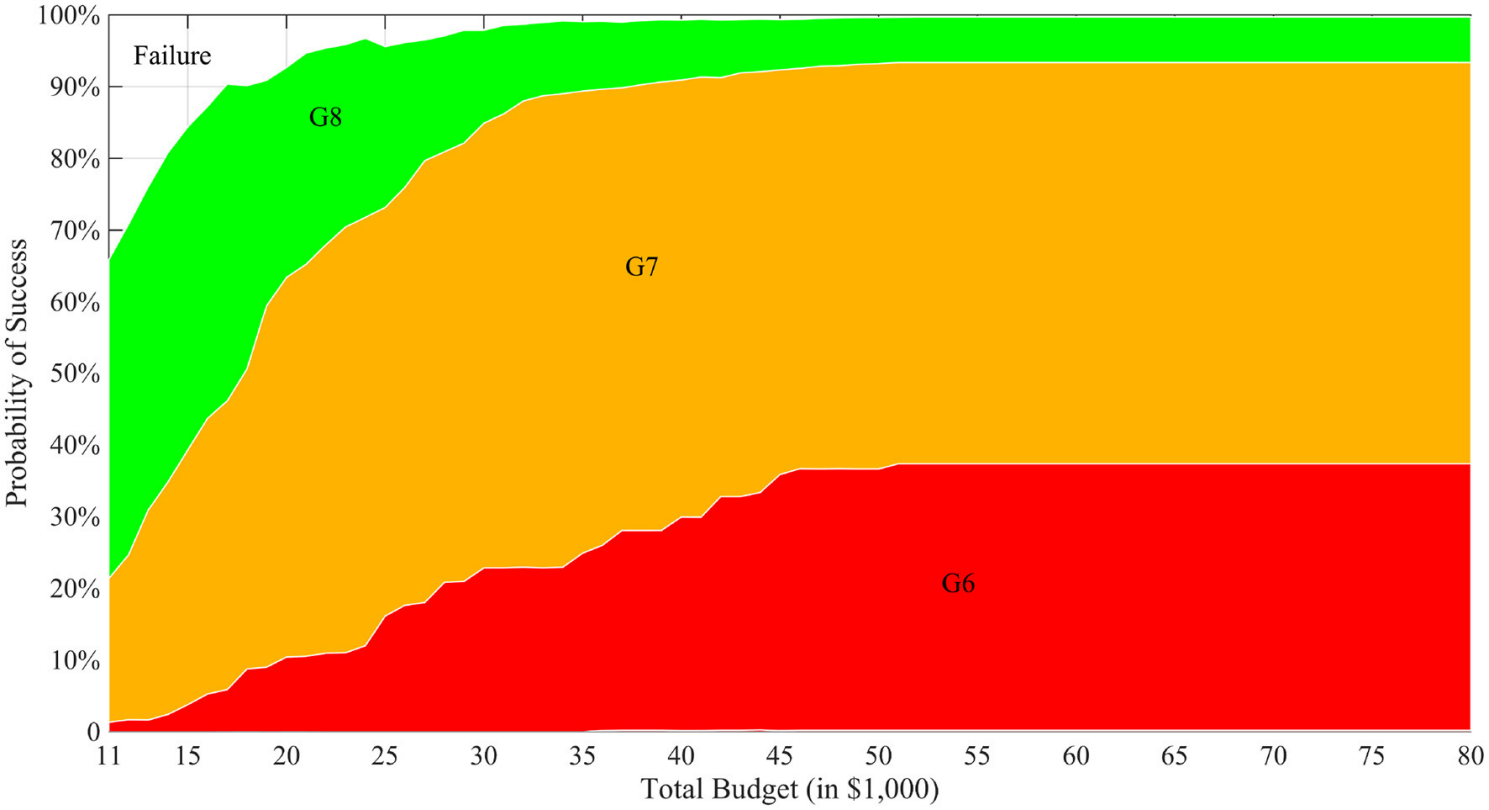
CTP graph with *T* = 8. In the figure, the horizontal axis is different total budget scenarios of the breeding project and the vertical axis represents a stacked histogram of the probabilities of reaching success at different generations. In the figure, “G*X*” label means that the breeding process successfully finishes in *X* generations and “Failure” means no ideal individual is produced when the budget or the time is depleted.

We first ran the simulation with varying levels of total budget for a static budget per generation and presented the results in the CTP framework associated with each total budget value in Figure 3. The horizontal axis shows the total budget (cost) for the MATI process, the vertical axis represents the stacked histogram of probabilities, and different layers with distinct colors indicate the number of generations (time) it takes to successfully complete the process. For example, when the total budget is $11,000, the project can successfully finish in 6, 7 or 8 generations with probability about 2, 20, or 44%, respectively. This project also has about 34% probability to fail. The figure also demonstrates the diminishing effect of increased budget to the performance of the process. From a commercial breeding perspective, this would enable an organization to estimate the cost and time-length needed for successful creating the desired progeny.

#### 3.2.2. Comparison With Static Resource Allocation Strategies

We demonstrate the improvement of optimal dynamic resource allocation over the static resource allocation using two random simulations, which are summarized in the following tables of figures, Tables 2, 3. Table 2 shows the result simulated using the static strategy with *K*^*t*^ = 400 for each generation *t*, whereas Table 3 shows the result from the MDP model. In both tables, the first column is the generation number. In the second column, at each generation, all the progeny produced in the simulation are put abreast to each other to form a wide rectangle and the width of the rectangle reflects the sample size. Here we use gray pixels to represent the desirable alleles whereas black pixels to represent the undesirable alleles. Those individuals highlighted by white are the selected breeding parents and several ideal individuals are produced at the last generations. The third column of each table is the base 10 logarithm of PCV values of the selected breeding parents. The fundamental difference between these two resource allocation strategies is that the MDP model allows the decision maker to dynamically allocate the resources based on the outcomes from the previous generation. As a result, for the same amount of the total budget, the dynamic approach was able to produce the ideal progeny in the seventh generation, whereas the static strategy required an extra generation.

**Table 2:** Generations 2–8 of one random simulation run with fixed budget allocation.

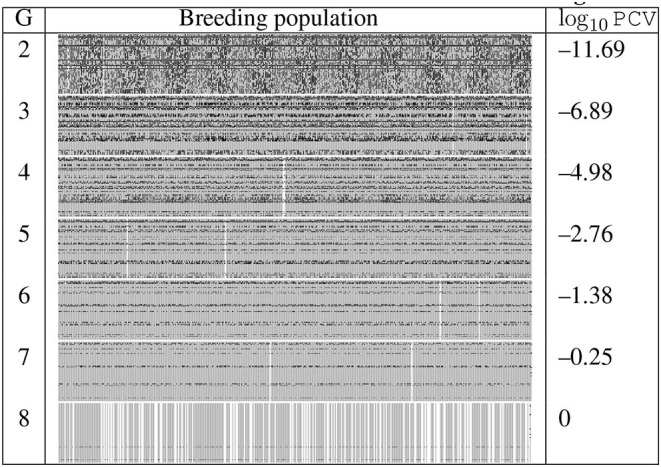

**Table 3:** Generation 2–7 of one random simulation run with MDP based budget allocation.

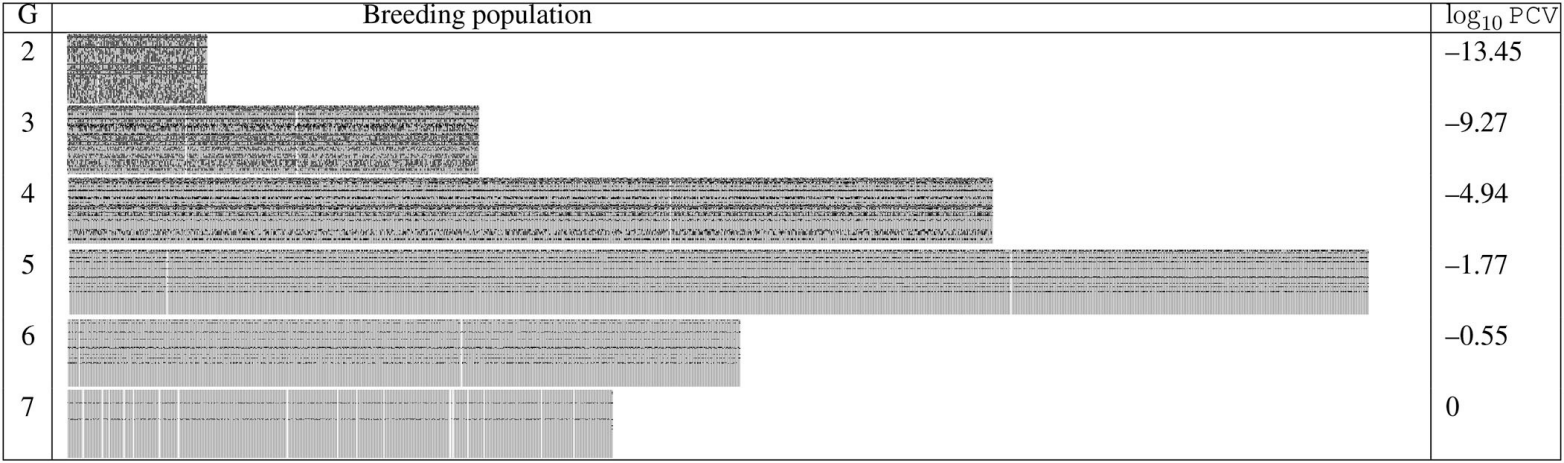

**Figure 4:**
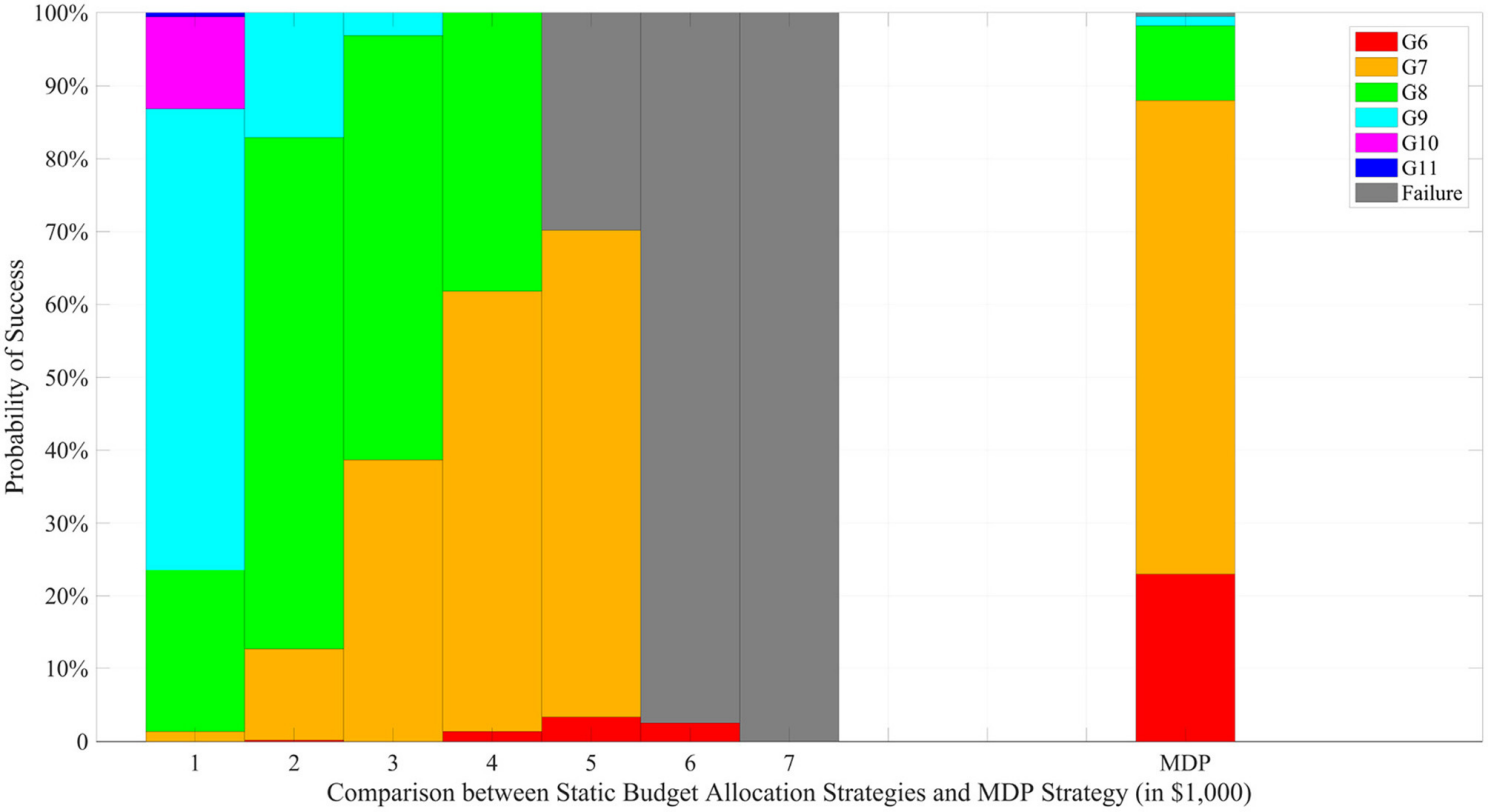
Comparison under a fixed total budget of $32,000. The left 7 stacked bars represent the static budget allocation strategies with different progeny number per generation while the last bar represents the MDP based strategy.

Figure 4 compares static and dynamic resource allocation strategies with respect to the CTP criteria for a fixed total budget of $32,000. We considered seven different static strategies, in which a fixed number of progeny (ranging from 100 to 700 with an increment of 100) are produced in each generation. A total of 500 simulation repetitions were conducted for the seven static strategies and the dynamic strategy, and the histograms of the terminal generations are compared in the figure. When a small number of progeny are produced, the static strategy takes more time resources to complete the project; when a large number of progeny are produced, on the other hand, the static strategy risks depleting the total budget before successful completion. For instance, the 600-strategy produces 600 progeny in each of generations 1–5 and only 200 progeny in generation 6 with a fixed total budget of $32,000. For such strategy, the success rate of achieving the ideal target in generation 6 is <5%. In contrast, the dynamic strategy has the flexibility to adjust the amount of resource allocation based on the outcome of the previous generation and is more likely to achieve successful completion within a shorter amount of time.

#### 3.2.3. Optimal Total Budget and Budget Allocation

**Figure 5:**
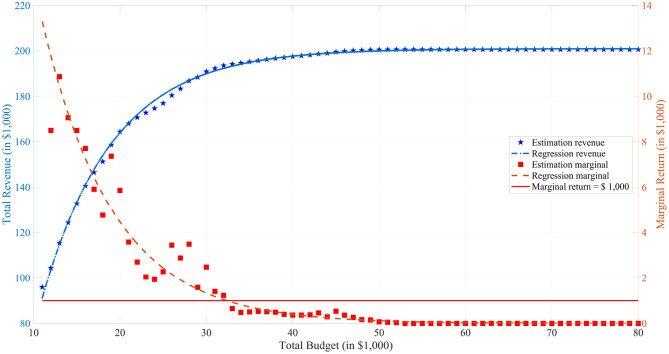
Profits and Budgets. In the figure, the blue pentagrams represent the estimation results from simulations and the blue curve represents a nonlinear regression with model *y* = *a*_1_ + *a*_2_ × exp(*a*_3_*x*) for the estimation. The red squares represent the difference between the adjacent estimations and the red curve represents the derivative of the expected total revenue curve. The red horizontal line is the marginal return is equal to one unit increment of total budget, which is $1,000.

Figure 5 enables plant breeders to determine the optimal total budget for the MATI project based on cost-benefit analysis. The blue curve represents a regression line on the estimated total revenue, referring to the blue axis on the left. The red curve represents a regression line on the estimated marginal return, which is the derivative of the total revenue, referring to the red axis on the right. This red curve illustrates the relation between the investment on the total budget and the relative gain on the total revenue. The optimal total budget, approximately $32,000, is achieved where the marginal revenue intersects with $1000, which is the unit increment of the total budget. Before the optimal budget, every extra unit total budget investment brings more return on the total revenue. However, after this point, the increment on the total revenue is comparatively less with the unit total budget increment.

**Figure 6:**
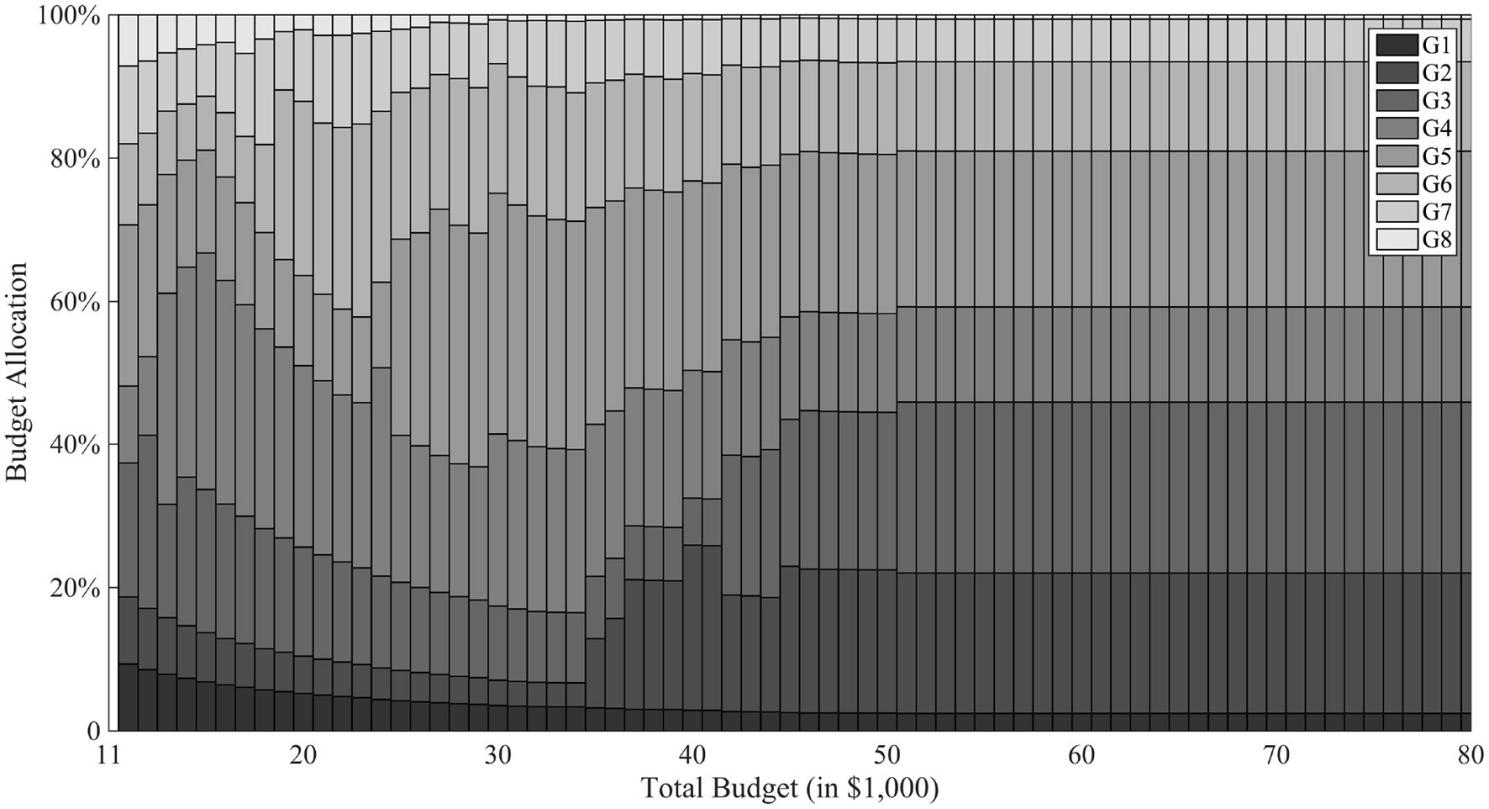
Budget allocation with *T* = 8. In the figure, the horizontal axis is different total budget scenarios of the breeding project and the vertical axis represents the proportion of budget allocated in different generations. Different gray scale are used for different generations.

Figure 6 breaks down the cost allocation to different generations for varying levels of total budget. When total budget is less than the optimal level, the model tends to allocate unproportionately higher percentages of budget to early generations, in order to produce enough progeny and preserve genetic diversity for future genetic gains. When total budget exceeds the optimal level, resource allocation to different generations becomes stable. Meanwhile, the model tends to allocate relative more resources on generation 2 and 3 to push the process to succeed in generation 5. However, if it is not finished in 5 generations, the model allocates a second push in generation 6 to pursue a quick success. In general, the model focuses on dynamic balance of both budget and time resources. After G1, the model tends to allocate higher budget in G2 and G3 to create variability; G4 requires less budget but a little time for favorable recombinations to happen; G5 gives a final push for the “lucky” progeny to succeed in G6 and subsequent generations.

## 4. Conclusions

In this paper, we addressed the issue of optimal resource allocation in a MATI process using a Markov decision process model, and made connections to the importance of optimizing this process for a commercial organization. Simulation experiments suggested that the proposed dynamic resource allocation method greatly improves the efficiency of the MATI process. Due to the assumptions made in the problem definition and model construction, the proposed model is by no means the best possible solution to the proposed problem, but this can be seen as a potential efficiency improvement on the traditional MATI process. Future research effort is needed to explore other definitions of the state space and action space to further improve the effectiveness of the model.

Estimating the cost and revenue function is a possible research topic for further discussion, as well. Plant breeding organizations have their own forecasting models about the market value of a certain genotype as well as its revenue associated with time when it is delivered to the market. Thus, the research on the discussion about cost and revenue functions may reveal more economic discoveries about the trait introgression problem and inspire further analysis.

Another fruitful research topic will be applying more advanced artificial intelligence techniques into such research problems. In our model, simplifying assumptions were made to reduce the problem dimension to a relatively small scale with only a few actions and states and finite time horizon. However, as studied in Hospital et al. (2000), different selection intensity or the number of parents selected for each generation could make this resources allocation challenge more comprehensive and complex. At the same time, relaxing the problem to allow multiple donors is challenging. Also, the assumption on independent crossovers could be changed for a more comprehensive analysis. At the same time, it would be a meaningful followup study to relate and compare with the gene-stacking algorithm in De Beukelaer et al. (2015), in which the population size was determined by a statistical formula. In order to solve such problems under fewer assumptions and higher dimensions, more powerful modeling and solution techniques, such as reinforcement learning will be necessary to deal with the uncertainty and complexity of the MATI process to discover more efficient strategies.

## Data Availability Statement

The raw data supporting the conclusions of this article will be made available by the authors, without undue reservation.

## Author Contributions

YH and LW conceived of the presented idea and developed the theory and performed the computations. JC and WB provided guidance on modifications according to domain knowledge. JC, HP, and WB verified the analytical methods and results. All authors discussed the results and contributed to the final manuscript.

## Conflict of Interest

The authors declare that the research was conducted in the absence of any commercial or financial relationships that could be construed as a potential conflict of interest.
