# Supplementary material for: Dynamic Programming for Resource Allocation in Multi-Allelic Trait Introgression
Source: Front Plant Sci. 2021 Jun 18;12:544854. doi: 10.3389/fpls.2021.544854 (PMC8253225; doi:10.3389/fpls.2021.544854)
Supplement: Supplementary file 1 [file Data_Sheet_1.PDF]

	C1	C2	C3	C4	C5	C6	C7
M1	0.2725	0.2075	0.0569	0.0860	0.1414	0.0791	0.0126
M2	0.2649	0.1957	0.0759	0.1362	0.1693	0.1529	0.2951
M3	0.2148	0.0692	0.1452	0.1983	0.0285	0.3210	0.3044
M4	0.1262	0.1004	0.1037	0.0874	0.0875	0.1823	0.2654
M5	0.2705	0.1570	0.3078	0.2009	0.2670	0.1737	0.0329
M6	0.1776	0.0768	0.1434	0.2371	0.0097	0.0772	0.0873
M7	0.1169	0.2814	0.0616	0.0739	0.3096	0.1630	0.1118
M8	0.3130	0.0649	0.3016	0.0391	0.2434	0.2080	0.2266
M9	0.2920	0.5000	0.3266	0.0989	0.1629	0.2264	0.0455
M10	0.5000		0.1463	0.5000	0.5000	0.1318	0.2404
M11						0.1225	0.5000
M12						0.5000	

C8	C9	C10
0.2179	0.0659	0.0610
0.1647	0.0102	0.0800
0.2597	0.2480	0.2955
0.2383	0.1667	0.0096
0.3012	0.1600	0.1633
0.2970	0.3016	0.0560
0.1114	0.2033	0.3262
0.5000	0.2059	
	0.2865	
	0.2685	
	0.5000	
